# Effective connectivity during visual processing is affected by emotional state

**DOI:** 10.1007/s11682-014-9326-8

**Published:** 2014-10-23

**Authors:** Miroslaw Wyczesany, Tomasz S. Ligeza, Szczepan J. Grzybowski

**Affiliations:** Psychophysiology Laboratory, Institute of Psychology, Jagiellonian University, Ingardena 6, PL-30060 Kraków, Poland

**Keywords:** Visual perception, Attention, Mood, Effective connectivity, Directed transfer function

## Abstract

The limitations of our cognitive resources necessitate the selection of relevant information from the incoming visual stream. This selection and prioritizing of stimuli allows the organism to adapt to the current conditions. However, the characteristics of this process vary with time and depend on numerous external and internal factors. The present study was aimed at determining how the emotional state affects effective connectivity between visual, attentional and control brain areas during the perception of affective visual stimuli. The Directed Transfer Function was applied on a 32-electrode EEG recording to quantify the direction and intensity of the information flow during two sessions: positive and negative. These data were correlated with a self-report of the emotional state. We demonstrated that the current mood, as measured by self-report, is a factor which affects the patterns of effective cortical connectivity. An increase in prefrontal top-down control over the visual and attentional areas was revealed in a state of tension. It was accompanied by increased outflow within and from the areas recognized as the ventral attentional network. By contrast, a positive emotional state was associated with heightened flow from the parietal to the occipital area. The functional significance of the revealed effects is discussed.

## Introduction

The processing of emotional stimuli is a complex and non-linear multi-stage process. On different levels, it is affected by both characteristics of the stimuli itself and numerous cognitive and affective factors such as expectations, cognitive set, previous experience, current mood, or learned strategies of emotional control (Corbetta and Shulman 2002). The influence is especially marked in the case of emotionally valenced content of high biological relevance, which potentially requires a fast and adequate response by the organism. Here we investigate how the current emotional state affects the brain areas involved in the perception of emotional content. We argue that connectivity analysis, which examines the brain in terms of a functional network, will bring us more understanding of the roles played by the areas considered.

It has been proven that stress and anxiety alter the recognition level of negative stimuli as well as the spatial characteristics of visual scene processing. Negative mood has been argued to improve the recognition of threatening content in a state of anxiety. This applies to both expected and unexpected stimuli – irrespective of their location in the visual field (Bishop et al. 2004; Gutiérrez and Gutiérrez Calvo 2011; MacLeod and Hagan 1992; Michalowski et al. 2009). The cost of such alteration in perception characteristics is a simultaneous decrease in efficiency in responding to peripheral, non-emotional stimuli (Rossi and Pourtois 2012). Also, a positive mood is known to affect the spatial scope of the visual field. Most data show a tendency to expand spatial attention (Wadlinger and Isaacowitz 2006). However contrary results can also be found (Vanlessen et al. 2013). The inconsistencies suggest the possible impact of other, uncontrolled factors.

The influence of mood is also observed during the visual processing of symbolic, verbal stimuli so that recognition speed and memory performance can be enhanced when reading emotional words that are congruent with the current affective state. Some reports, however, have suggested that this effect remains highly specific and only applies to congruence in discrete emotional states (Flor et al. 1997; Kiefer et al. 2007; Niedenthal and Setterlund, 1997, 1994). Late ERP effects characterized by an increase in the late positivity potential (LPP), or a decrease in the N400 component, in response to positive words may be attributed to the “default” positive mood during typical laboratory conditions and could also reflect the mood congruency effect (positivity offset; Herbert et al. 2006; Herbert et al. 2008; Kiefer et al. 2007). On the other hand, very early effects related to sensory analysis (within 100 ms of word onset) could point to negativity bias, with higher responses evoked by negative words, but only under more arousing circumstances, i.e. in an emotional context (Grzybowski et al. 2014).

Despite the boosting of initial phases of perceptual processing in a negative state, overall behavioral performance can still deteriorate. The higher cognitive functions, from the late stages of perception up to executive processes, can be altered in negative mood (Li et al. 2013), or even disrupted during serious stress or anxiety, which results in lowered performance of goal-related activity due to possible depletion of available resources (Shackman et al. 2011).

To understand affective modulation of perception, it is important to identify what particular stages of processing are affected, since they collectively make up the observed effects. In terms of attention, selective attenuation or enhancement is often considered as a gating mechanism. However, attention is not a unitary phenomenon and two general systems can be distinguished. The first one, the dorsal attentional network (DAN), focuses on the information that is relevant to the current task or goal-related requirements. It represents top-down control over the perceptual system and is responsible for both spatial and feature-related attention. Anatomically, it is identified as the dorsal fronto-parietal network and is composed of bilateral frontal eye fields (FEF) and intra-parietal sulci with superior parietal lobules (IPS/SPL; Corbetta and Shulman 2002; Serences et al. 2005). This network can exert specific control over the perceptual system, from primary to association visual areas in striate and extrastriate cortices (Ruff et al. 2008). Another system, known as the ventral attentional network (VAN), controls for stimuli-driven shifts of attention. Its main function is reorienting the resources toward behaviorally relevant stimuli even when they are unexpected or unattended. It relies on a distributed network located mostly in the right hemisphere: part of the middle and inferior frontal gyrus (MFG/IFG), anterior insula (aIns) and temporoparietal junction (TPJ). The latter is thought to provide an interface with the dorsal network. The ventral system is also influenced by the subcortical limbic structures (amygdala), which can also contribute to the regulation of perceptual sensitivity (Corbetta et al. 2008; Kotani et al. 2009; Salmi et al. 2009).

The two attentional systems are inseparably bound, while providing prioritized processing of relevant information. Current models assume inhibition of the ventral system by the dorsal during task-engagement, which prevents unnecessary distraction by salient but non-relevant stimulation. On the other hand, the ventral system is important for reorienting the dorsal system when important changes in the environment occur (Frank and Sabatinelli 2012; Todd et al. 2005). Yet because investigations into the precedence of neural activations in both systems have yielded inconsistent results, the details and causality of this process are still not clear (Corbetta et al. 2008; Fox et al. 2006). It has also been proposed that the two systems act together to regulate the range of information entering conscious perception (Asplund et al. 2010).

A number of models have already been proposed to explain the causal relationships within the perceptual-attentional system and their role in mood-related changes in perception. It is possible that increased focus on emotional content during an anxiety state observed especially for negative stimuli is based on sensitization of the amygdala toward potential threat signals. Then, the amygdala could further influence the sensory cortex by increasing its activation directly – by neural re-entrant connections – and more generally via the locus coeruleus and norepinephrine neuromodulatory paths (Corbetta et al. 2008; Vuilleumier 2005). An alternative explanation based on the attentional control theory (Eysenck et al. 2007; Fiedler et al. 2003) points out that stress and anxiety facilitate the bottom-up, stimulus-driven system, which may transiently prevail over top-down control. Another possibility is a tonic increase of prefrontal control over the visual areas in a negative emotional state in order to enforce utilization of more resources for identifying potential negative stimuli (Vanlessen et al. 2013). The prefrontal influence is also suggested as a plausible mechanism for mood-related alterations in cognitive processes, including broadening of attentional scope in a positive emotional state (Rowe et al. 2007; Wadlinger and Isaacowitz 2006). Since these mechanisms can act in parallel, the alternative views need not be contradictory.

The existing models of perceptual control are based to a large extent on fMRI data, characterized by low temporal resolution and thus rather correlational in their nature. To step beyond these limitations, it would be beneficial to utilize methods that can determine the paths of neural information flow and thus allow inferences to be made about the causality of brain processes. In the present article, we investigate mood-related changes in effective connectivity when attending emotional pictures by means of Directed Transfer Function (DTF) applied on the EEG recording (Blinowska et al. 2004; Korzeniewska et al. 2003). This autoregressive method is based on Granger causality principles and provides an estimation of the amount and direction of information flow based on signals from multiple electrodes while controlling the familywise alpha level (Kamiński et al. 2001). The procedure was designed to quasi-simultaneously record brain activity and self-reported affective state while attending emotional pictures. The passive viewing context was chosen to minimize the interference of cognitive activity with the emotional state (Wyczesany et al. 2008). The subjects took part in two separate sessions, positive and negative, with mood induction by means of highly valenced stimuli to make the emotional state more variable across subjects (Westermann et al. 1996; Wyczesany et al. 2009). Self-assessment scores were then correlated with DTF values separately in both sessions. Because the analysis is carried out using the actual state of the subject as the independent variable, a design such as this – which includes direct assessment of the current affective state – allows for inference even when the mood induction is not fully successful.

It was hypothesized that a negative emotional state would be related to increased feedback from the prefrontal area to both attentional systems and this effect would be visible especially for negative pictures. This would reflect sensitization to threat-related content. Moreover, increased flow from the ventral to the dorsal attentional network (via the TPJ) was expected as a marker of facilitation of bottom-up processes and simultaneous inhibition of top-down attentional control associated with the increased role of stimuli salience. Finally, it was posited that negative stimuli – biologically relevant as they signal potential danger and thus require immediate responses – would reveal stronger effects than those observed for positive ones.

Additional computations were performed using prestimulus alpha power over the visual cortex to integrate the connectivity analysis with classic EEG methods. Its level during an idle period, just before the presentation of the stimulus, is thought to reflect the active top-down influence that modulates the sensitivity of the perceptual system (Dijk et al. 2008).

## Materials and methods

### Procedure

The procedure was compliant with the directives of the Helsinki Declaration (1975, revised 2000) and approved by the Ethical Committee of the Institute of Psychology, Jagiellonian University. Informed consent was obtained from all subjects. Forty-two undergraduate student volunteers (23 women; mean age 20.1, SD 1.7), participated in the study after signing a written consent form. All of them were medication-free with no reported history of neurological and psychiatric diseases – or of substance abuse – and had normal or corrected-to-normal vision. During the procedure, the subjects were seated in an air-conditioned soundproof cabin before a 24″ LCD monitor.

**Fig 1:**
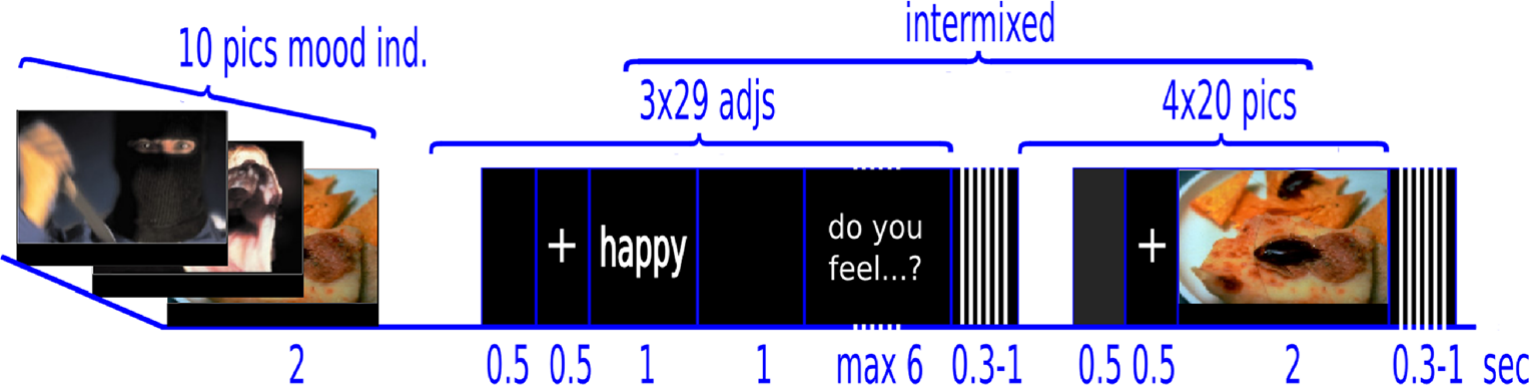
The time-course scheme of the procedure

There were two randomly sequenced sessions, one positive and one negative, which were determined by the valence of the slides displayed. Forty emotional pictures were chosen from the International Affective Picture System (IAPS) database (Lang et al. 1999). Half of them had a high negative rating (mean 2.1) and half had a high positive rating (mean 7.2). Both sets were matched for arousal (mean 5.7 and 5.6 respectively) and additional precautions were taken to equate them – where possible – in terms of content and complexity. Both sets thus included humans (seven photos depicted faces in the positive set and nine in the negative set), animals, landscapes, and food.^1^ Each session started with a presentation of ten pictures randomly chosen from the main set (full-screen, 2 s, ISI 0.5 s). They were primarily intended to influence the participants’ mood and were not analyzed in the connectivity computations that follow. The main section of the experiment in which the emotional stimuli were presented then began. The pictures were intermixed with adjectives (with a response scale provided) intending to measure the subjects’ current emotional state. The pictures and adjectives were presented in random order. Apart from the number of repetitions of each item, which was fixed, no additional constraints were set for the sequence of stimuli; there could be one or more pictures followed by one or more adjectives. The parameters for the picture presentation were as follows: 0.5 s blank screen, 0.5 s fixation cross, 2 s display time, ISI randomly 0.3 to 1 s, full-screen, each picture repeated four times, and 160 trials in total. As for the adjectives, 29 items taken from the Polish version of the UWIST Mood Adjective Checklist (UMACL; Matthews et al. 1990) were used. The adjectives were followed by a questionnaire-based inquiry – “Do you feel that way?” – with a four-point rating scale. Each adjective (black letters on a grey background, Liberation Sans font, 24p) was repeated three times and preceded by a blank screen interval (random length from 0.3 to 1 s) and a fixation cross (0.5 s). Positive and negative sessions were separated by a break. The procedure was then stopped and the subjects were free to leave the experimental room. This was intended to attenuate the mood induction effect from the previous session. The schema of the experimental procedure is depicted in Fig. 1.

### Data recording and analysis

EEG recording was carried out using a Biosemi Active Two device equipped with 32 active electrodes placed on a 10–20 headcap and four additional leads located above and below the right eye and in the external canthi of both eyes. The preprocessing was done using an EEG lab toolbox (Delorme et al. 2011), where the signal was off-line referenced to linked mastoids (recorded with two more electrodes) and filtered in a range of 1 Hz to 45 Hz with zero phase-shift filters. Two-second epochs (0 to 2 s relative to picture onset) were extracted. No oculomotor correction was applied in the case of DTF analysis; instead, segments including artifacts were rejected. First, to detect eye blinks, a 100 μV rejection threshold was set for all of the electrodes, including the one located over the right eye, where the blinks were most prominent. Next, to reveal significant eye movement artifacts (especially saccades), Independent Component Analysis was used to separate horizontal movement signals, which were identified using the criteria of scalp distribution and temporal characteristics (Jung et al. 2000). Trials in which the amplitude of the eye movement component exceeded 30 μV were rejected. Based on the Akaike information criterion (AIC), the autoregression model order for analysis was set to 7. A check was made before the connectivity analysis to see if the length of data remaining after the rejection procedure was sufficient for DTF calculations. The following formula was applied: W ≥10*(pM/N) where: W – required minimum window length in samples, p – model order, M – number of channels and N – total number of epochs for all trials within each valence condition (Korzeniewska et al. 2003). The criterion was fulfilled for all subjects. DTF calculations were made using Multar software (Department of Biomedical Physics, University of Warsaw; www.eeg.pl). Based on EEG montage brain atlases (Kaiser 2007; Okamoto et al. 2004), the electrodes corresponding to the following regions of interest (ROIs) were selected for connectivity analysis: right inferior frontal gyrus, IFG (VAN): F8; right temporoparietal junction, RTPJ (VAN): CP6; left/right intra-parietal sulci, IPS (DAN): CP1, P3, CP2, P4; striate/extrastriate visual cortex: O1, Oz, O2, PO3, PO4; left/right prefrontal cortex; and PFC: Fp1, AF3, AF4, Fp2. Since the DTF method estimates the information flow based on the phase difference between channels, it remains insensitive to the problem of volume conduction, as the electromagnetic fields propagate near-instantaneously with no phase differences between electrodes (Kamiński and Blinowska 2014). This allows for more precise location of underlying structures, with no additional signal processing (such as Laplace or beamforming; Nunez et al. 1997). Normalized DTF values for all pairs of electrodes between the ROIs were then calculated for the frequency important in attentional processes (beta 14 Hz-25 Hz), which is especially significant in the visual domain (Gola et al. 2013; Kamiński et al. 2012; Kus et al. 2008).

To control the quality of autoregressive model fitting, the residual noise matrices were determined for all subjects. The distributions of the DTF values obtained were checked to identify and reject possible extremes, which were defined as falling below Q1-1.5*IQR or above Q3 + 1.5*IQR (Q – quartile; IQR – interquartile range; Tukey 1976; Wyczesany et al. 2014).

To determine the valence of the subjects’ mood, the self-report scores were calculated separately for both sessions according to the original UMACL instructions. This yielded three dimensions: Energetic Arousal (EA: a measure of the organism’s overall arousal level), Tension Arousal (TA: a negatively valenced state of tension, worry, and anxiety) and Hedonic Tone (HT: a pleasant and enjoyable state).

Next – after ensuring normal distributions of data by means of a series of Kolmogorov-Smirnov tests – the relationships between the subjective scales and DTF values were estimated separately in positive and negative sessions using Pearson’s r correlations. Hence, flow increases or decreases refer to positive or negative correlations respectively, with the subjective scales given observed for the extracted epochs. Finally, because there were multiple comparisons involved, the False Discovery Rate (FDR) procedure (Benjamini and Hochberg 1995) was applied for all flows between the ROIs considered to correct significance levels.

The one-second epochs directly preceding the pictures were extracted for prestimulus spectral analysis. They were corrected for ocular artifact by subtracting ICA components classified as eye-movements and blinks based on their spatial and temporal characteristics (Jung et al. 2000). The spectral power for the alpha band (8 Hz-12 Hz) was calculated using the multitaper method (three orthogonal Slepian tapers) on the electrodes over the visual cortex ROI. Spearman correlations between alpha power and DTF values, measuring top-down information transfer from the left and right prefrontal cortex (Fp1, AF3 / Fp2, AF4) to the visual area (PO3, O1, Oz, O2, PO4), were calculated separately for the negative and positive sessions.

## Results

The sessions differed significantly in terms of all three subjective dimensions, which were assessed using repeated measures t-tests. The direction of changes confirmed the efficiency of the mood induction, which affected the entire duration of each session. When compared to the positive pictures, the negative ones were associated with higher tension (TA) and lower energetic arousal (EA), as well as with more negative valence (lower HT scores; see Table 1).

**Table 1:** Group means of subjective scores with differences between sessions

	negative session mean	positive session mean	change (NEG-POS)	t	df	significance (p)
Energetic Arousal (EA)	24.13	25.18	−1.05	−2.15	41	0.038
Tension Arousal (TA)	19.86	15.01	4.85	6.93	41	<0.001
Hedonic Tone (HT)	22.79	29.93	−7.14	−8.75	41	<0.001

**Fig. 2:**
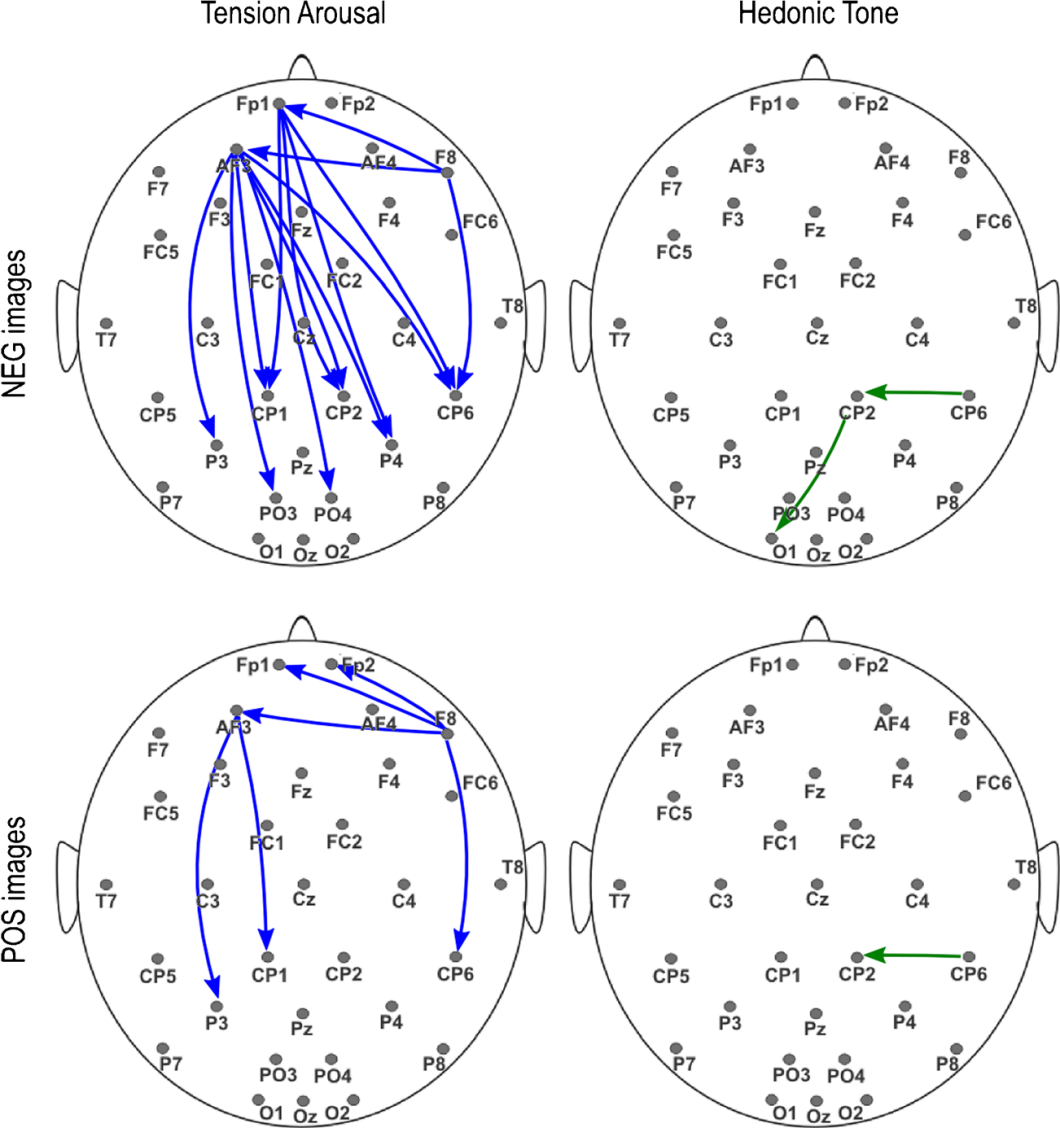
Directions of information flow (normalized DTF values) which significantly correlated with the self-report scores in the β freq band

**Fig. 3:**
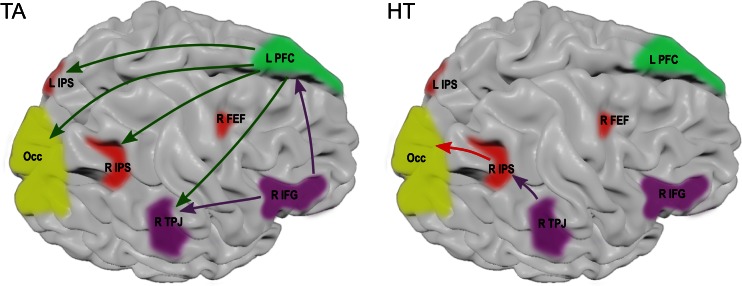
Projection of observed effects onto the brain surface with location of putative structures involved. Negative session effects are shown. Solid lines denote positive correlations. *L PFC* left prefrontal cortex, *R IFG* right inferior frontal gyrus, *R FEF* right frontal eye field, *TPJ* temporoparietal junction, *IPS* intraparietal sulcus, *Occ* occipital visual cortex

The autoregression model fitting for the EEG signal was good enough in all subjects, i.e. mean RV values did not exceed 13 %. The most significant correlations between subjective scales and connectivity measures were found for the TA scale; in all cases the affected sources increased their outflow with elevated tension. In the negative session, the following directions of flow showed significant effects: from Fp1 to CP1, CP2, P4 and CP6; from AF3 to CP1, CP2, P3, P4, CP6, PO3 and PO4; and from F8 to Fp1, AF3 and CP6 electrodes. In the positive session, the number of significantly affected flows was lower: from AF3 to CP1 and P3, and from F8 to Fp1, Fp2, AF3 and CP6 electrodes. The effects of the positive valence (higher HT scores) were seen as increased flow from the CP6 to CP2 electrode for both sessions, and from the CP2 to the O1 electrode only in the negative session. Detailed correlation coefficients with corresponding p-values (both raw and FDR-adjusted) are shown in Tables 2 and 3, and are visualized on the scalp surface in Fig. 2. For further interpretation of the results, the significant effects were mapped onto the putative underlying brain structures using the predefined ROIs (see Fig. 3 for the negative session). In terms of ROIs, the observed effects of the TA scale included the following flows: In the negative session – from the left PFC to the bilateral IPS and visual area, and from the right IFG to the left PFC and the right TPJ; In the positive session – from the left PFC to the left IPS, and from the right IFG to the prefrontal area as well as to the right TPJ. The effects of the HT scale included increased flow from the right TPJ to the right IPS in both sessions, as well as from the right IPS to the visual area in the negative session.

**Table 2:** Significant correlations between DTF values and Tension Arousal scores observed in the β frequency band

Tension Arousal effects						
	negative pictures			positive pictures		
flow direction	correlation (r)	raw significance (p)	FDR-corrected significance (p)	correlation (r)	Raw significance (p)	FDR-corrected significance (p)
LPFC → IPS						
AF3 → CP1	0.31	0.02	0.03	0.43	0.00	0.02
AF3 → CP2	0.46	0.00	0.01	–		
AF3 → P3	0.27	0.04	0.05	0.25	0.05	ns
AF3 → P4	0.33	0.02	0.03	–		
Fp1 → CP1	0.31	0.03	0.03	–		
Fp1 → CP2	0.37	0.01	0.03	–		
Fp1 → P3	–			–		
Fp1 → P4	0.34	0.02	0.03	–		
RIFG → PFC						
F8 → AF3	0.29	0.03	0.05	0.28	0.04	0.05
F8 → Fp1	0.31	0.03	0.05	0.34	0.01	0.03
F8 → AF4	–			–		
F8 → Fp2	–			0.33	0.02	0.03
LPFC → RTPJ						
AF3 → CP6	0.36	0.01	0.03	–		
Fp1 → CP6	0.33	0.02	0.03	–		
AF4 → CP6	–			–		
Fp1 → CP6	–			–		
LPFC → Occ						
AF3 → PO4	0.46	0.00	0.02	–		
AF3 → PO3	0.39	0.01	0.03	–		
AF3 → O1	–			–		
AF3 → O2	–			–		
AF3 → Oz	–			–		
Fp1 → PO4	–			–		
Fp1 → PO3	–			–		
Fp1 → O1	–			–		
Fp1 → O2	–			–		
Fp1 → Oz	–			–		
RIFG → RTPJ						
F8 → CP6	0.38	0.01	0.01	0.28	0.04	0.04
P4 → O1	–			–		
P4 → Oz	–			–		
P4 → O2	–			–

**Table 3:** Significant correlations between DTF values and Hedonic Tone scores observed in the β frequency band

Hedonic Tone effects						
	negative pictures			positive pictures		
flow direction	correlation (r)	raw significance (p)	FDR-corrected significance (p)	correlation (r)	raw significance (p)	FDR-corrected significance (p)
RTPJ → IPS						
CP6 → CP1	–			–		
CP6 → P3	–			–		
CP6 → CP2	0.34	0.01	0.05	0.38	0.01	0.05
CP6 → P4	–			–		
RIPS → Occ						
CP2 → O1	0.31	0.02	0.05	–		
CP2 → Oz	0.37	0.01	ns	–		
CP2 → O2	–			–		
P4 → O1	–			–		
P4 → Oz	–			–		
P4 → O2	–			–

Significant negative correlation between the alpha power and average DTF from the left prefrontal cortex to the visual area was observed in the negative session (Spearman’s rho = −0.45, *p* = 0.005), while the positive one revealed no significant relationships.

## Discussion

We investigated the mood-related changes of connectivity between the brain’s perceptual, attentional, and prefrontal control centres. We assumed that their effective connectivity could underlie the dynamic modulation of perception and the processing of emotional stimuli, which are associated with the current affective state. The results show that the level of Tension Arousal (TA), as a scale strongly linked to negative valence, turned out to be the most influential factor affecting the strength of connectivity. Some results for Hedonic Tone (HT), which, on the other hand, is strongly linked to positive valence, were also observed. In contrast, Energetic Arousal (EA) did not yield any significant results. Since the EA scale is associated mostly with the energetic and not the valence aspect of the emotional state (Wyczesany et al. 2010), the null result suggests that the valence may be the main factor influencing information flow, which is discussed further elsewhere in the text.

As expected, mood-related changes in effective connectivity were more prominent in the negative session. This is not surprising, since swift and adequate responses to external signals are more critical for the organism in conditions that carry a potential threat. This confirms other reports that have shown heightened coupling of brain regions (Moratti et al. 2011), or increases in brain activations, during perceptual tasks observed in a state of tension when compared to those observed in relaxed and safe conditions (Dalton et al. 2005). Although the effects seen in the positive session were usually weaker, it is worth noting that many of them were apparently a subset of those visible during the negative session. This supports the claim that the relationships observed between the current emotional state and the effective connectivity can be considered as a more general pattern related to emotional arousal, which are therefore not limited to specific experimental conditions.

A massive intensification of information flow from the left prefrontal cortex, related to emotional tension, confirmed our hypotheses. The affected areas included those putatively corresponding both to attentional systems (DAS: both IPS under the CP1, P3, CP2, P4 electrodes; VAS: right TPJ under the CP6 electrode) as well as to the visual cortex (O/PO electrodes). This vast increase in signal propagation can be interpreted as a heightened top-down influence enforced on the attentional and perceptual systems. Such a pattern could possibly underlie mood-related sensitization to potential threat signals, which can be referred to as “hyper-vigilance”. Indeed, the strengthening of top-down connections is suggested as a phenomenon accompanying the prioritized processing of affective content (Lee et al. 2010).

This also confirms the fMRI connectivity data, which shows attention-dependent changes in coupling between the visual cortex and the IPS (Büchel and Friston 1997; McLaughlin et al. 2009). Connectivity between the occipital and prefrontal areas is also observed to increase during involvement in demanding perceptual tasks (Rowe et al. 2005). The causal role of the left dorsolateral PFC in intensifying the processing of negative stimuli has also been demonstrated in a TMS study (D’Alfonso et al. 2000), where dorsolateral PFC stimulation resulted in disruption of attention toward negatively valenced faces.

It is also important to note that the connectivity results were supported by the prestimulus alpha power analysis. This activity – recorded over the visual cortex during the period of anticipation of visual stimuli – can be considered a reverse marker of the top-down influence. It was shown to covary with the level of perceptual sensitivity and ability to discriminate visual stimuli and reflect the mechanism of visual input regulation (Dijk et al. 2008; Min and Herrmann 2007; Romei et al. 2010). The strong negative correlation between the pre-stimulus alpha and the prefrontal-visual connectivity confirms our interpretation relating this top-down link with modulation of the early, perceptual stage of visual processing. It is worth pointing out that this relationship was observed only in the negative session, which shows the importance of this modulatory process in the case of negative, biologically relevant stimuli.

Although we focus here on the automatic boosting of attention, an alternative interpretation cannot be ruled out. As can be seen, the prefrontal effects were limited to the left side. Indeed, some data have shown lateral specialization of the dorsolateral part of the PFC (DLPFC) as a control center. It has been suggested that the left DLPFC plays a dominant role in continuously monitoring the demands of the current situation and imposing automatic control over the attentional systems (MacDonald et al. 2000; Stuss et al. 2001; Vanderhasselt et al. 2009). However, the DLPFC regions are also known to be crucial for affective control (Buhle et al. 2013). The right DLPFC (compared with the left DLPFC) has been linked more frequently with the regulation of emotional processing in highly demanding conditions, such as in the presence of high intensity stimuli, in which additional cognitive control resources are required (Ochsner et al. 2012; Silvers et al. 2014). The lack of right DLPFC effects (compared with the left DLPFC) may therefore be explained by the passive procedure, where subjects were not engaged in any explicit, demanding task. Conversely, the activity in the left DLPFC could be related to an implicit form of affect regulation. Such a process could be initiated by the simple registration of sensory inputs and could take place without awareness and down-regulate the negative affect (Gyurak et al. 2011). There are data showing that the state of anxiety (which is closely related to tension arousal in our study) lowers the threshold for evoking a fear response to threatening stimuli which, on the other hand, can initiate affective control mechanisms (Bishop 2008). Strong emotional tension (especially in the negative session) could provoke mechanisms for mood modulation to some extent and thus additionally augment implicit down-regulation of affect (Tupak et al. 2014).

This is not, however, contradictory to our main line of reasoning, which points to modulatory influences on the perceptual/attentional system. The mood-related control of attention and possible down-regulation of affect are known to be partly related to each other (Okon-Singer et al. 2007), and these mechanisms could overlap during the negative session (it is unlikely that the positive session would have elicited significant down-regulation of affect). While mood-related control of attention could be seen in the positive and negative sessions, down-regulation of affect may represent additional outflows from the PFC regions in the negative session. In summation, these two facts support the importance of automatic attention control, which we propose as underlying our results.

Self-reported tension was also associated with an increased flow from the areas recognized as the ventral attentional network (F8 electrode mapped onto the right IFG) to certain other PFC regions. This was also accompanied by strengthening of connectivity with the right TPJ (located under the CP6 electrode). Importantly, these effects were visible for both sessions, which indicates that they may appear prior to full stimuli evaluation as a tonic sensitization to potential changes in the environment. Indeed, the role of the right IFG in modulating different features of visual attention by influencing the occipital and parietal areas has been widely shown (Corbetta and Shulman 2002; Hedden and Gabrieli 2006; Zanto et al. 2011). In line with these observations, this effect can be attributed to the increased involvement of the VAN observed at the connectivity level.

The predicted link between the ventral and task-oriented networks was identified as a specific and spatially limited flow from the CP6 electrode (over the right TPJ) to the CP2 electrode (over the right IPS). Thus the hypothesis that the path of communication between both attentional networks involves the right TPJ as an interface (Serences et al. 2005; Vossel et al. 2013) received some support. However, contrary to this hypothesis, the flow of the link under consideration was found to decrease in a negative emotional state when what was expected was the inhibition of the task-oriented attentional network by the stimulus-driven one. This reversed relationship leaves room only for speculative interpretations. Hence, it is possible that the observed flow represents a facilitatory instead of an inhibitory influence from the ventral network. Such an explanation could be supported by the data showing that behavioral, goal-directed performance related to expectation of visual cues is improved with decreased Granger influence from the right TPJ to the right IPS, which does indeed suggest the inhibitory character of this connection (Wen et al. 2012).

Positive valence of the emotional state (increased HT scores) was found to be associated with a heightened flow from the electrodes over the right IPS to the occipital area. This effect possibly relates to the extension of the spatial scope of attention in a positive mood, which is accompanied by a simultaneous decrease in sensitivity to negative signals. Some data suggest the causal role of the right IPS in regulating this trade-off by strengthening the top-down spatial features of attention (Oliveri et al. 2010). Our data support this view, by showing increased flow from the parietal area to the visual cortex in more positive affect.

### Conclusions and limitations

As can be seen, the DTF method proved to be a valuable tool which allowed us to ascertain the direction and changes of information flow based on EEG data. The data presented shed more light on the interaction between perceptual, attentional, and control brain areas and the emotional state as a factor influencing the process of visual perception. Although both the positive and negative conditions produced similar observations, the latter revealed more prominent effects.

We showed a tension-related increase of information outflow activity located in the left lateral prefrontal area. This effect was interpreted as sensitization driven from the top-down. It was found to involve a simultaneous influence on multiple cortical areas and thus to affect different stages of information processing. It was also supposed that negative conditions recruited some additional mechanisms for implicit, affective down-regulation, which could add to the perceptual/attentional control activity. In conclusion, our result supports the role of the prefrontal cortex as a crucial area for the integration of cognitive functions and the modulation of the entire attentional/perceptual system. Reported tension was also associated with increased connectivity within and from the areas recognized as the dorsal attentional network (RIFG and RTPJ). This would seem to be a plausible neural mechanism underlying the sensitization of the stimuli-driven attention. On the other hand, positive mood (HT scale) was related to increased flow from the right parietal area to the visual cortex, which could underlie changes in attentional scope.

Most importantly, we managed to demonstrate that the current mood, as measured by self-report, is a factor that affects the patterns of effective cortical connectivity. A multiple-item checklist tool was chosen for use in the study, which was intended for more implicit mood measurement, where the researcher’s intentions are not easily disclosed. The intermixing of emotional stimuli with adjectival items could be less susceptible to the influence of demand characteristics, since the consideration of possible experimental expectations in subjects is limited when they are focused on a pending procedure. These factors suggest that the accuracy of such quasi-simultaneous measurement is possible and better than a retrospective check (Hurlburt and Heavey 2001; Thomas and Diener 1990). It is known on the other hand that even simple cognitive tasks, such as rating or labelling emotional stimuli, can reduce the reactivity of limbic structures (Lieberman et al. 2007; Taylor et al. 2003). We argue, however, that because mood assessment was separated from emotional stimuli in our procedure this effect was minimized.

Some other limitations and future directions should also be mentioned. Firstly, the limited spatial accuracy of EEG should be borne in mind when interpreting the results. In this way the proposed correspondence between electrodes and cortical areas should be treated as putative but not certain. Secondly, the indirect inference about attentional characteristics was based on existing theoretical models that link the emotional state with changes in perception. Further verification of the results is recommended in future studies, where different task demands and attentional manipulation can be applied.

## Footnotes

1 The IAPS picture numbers for the positive set were: 1710, 1722, 2208, 2209, 2345, 4599, 4641, 4650, 5270, 5450, 5470, 5623, 7220, 7230, 7270, 7502, 8300, 8420, 8496, 8500; and for the negative set: 2710, 3015, 3220, 3230, 3301, 3350, 3550, 6570, 7380, 9253, 9300, 9400, 9405, 9420, 9500, 9520, 9560, 9570, 9571, 9920.
